# Adsorption of Trace Estrogens in Ultrapure and Wastewater Treatment Plant Effluent by Magnetic Graphene Oxide

**DOI:** 10.3390/ijerph15071454

**Published:** 2018-07-10

**Authors:** Xianze Wang, Zhongmou Liu, Zhian Ying, Mingxin Huo, Wu Yang

**Affiliations:** 1 Science and Technology Innovation Center for Municipal Wastewater Treatment and Water Quality Protection, Jilin Province, Northeast Normal University, Changchun 130117, China; wangxz940@nenu.edu.cn (X.W.); huomx097@gmail.com (M.H.); 2 Engineering Lab for Water Pollution Control and Resources Recovery, Jilin Province, Northeast Normal University, Changchun 130117, China; 3 School of Environment, Northeast Normal University, Changchun 130117, China; liuzm338@nenu.edu.cn (Z.L.); yingza451@nenu.edu.cn (Z.Y.)

**Keywords:** estrogens, magnetic graphene oxide, adsorption, wastewater treatment plant effluent

## Abstract

In the current study, graphene oxide, Fe^3+^, and Fe^2+^ were used for the synthesis of magnetic graphene oxide (MGO) by an in situ chemical coprecipitation method. Scanning electron microscopy, transmission electron microscopy, Fourier transform infrared spectroscopy, and X-ray diffraction were used to characterize the well-prepared MGO. The prepared MGO was used as an adsorbent to remove five typical estrogens (estrone (E1), 17β-estradiol (E2), 17*α*-ethinylestradiol (17α-E2), estriol (E3), and synthetic estrogen (EE2)) at the ppb level from spiked ultrapure water and wastewater treatment plant effluent. The results indicated that the MGO can efficiently remove estrogens from both spiked ultrapure water and wastewater treatment plant effluent in 30 min at wide pH ranges from 3 to 11. The temperature could significantly affect removal performance. A removal efficiency of more than 90% was obtained at 35 °C in just 5 min, but at least 60 min was needed to get the same removal efficiency at 5 °C. In addition, an average of almost 80% of the estrogens can still be removed after 5 cycles of MGO regeneration but less than 40% can be reached after 10 cycles. These results indicate that MGO has potential for practical applications to remove lower levels of estrogens from real water matrixes and merits further evaluation.

## 1. Introduction

China has the largest population in the world and has been facing a serious water crisis and water pollution problem in the past decades. According to the annual statistical yearbook for urban construction, 4.8 × 10^10^ m^3^ of wastewater was discharged in 2016 and more than 93% of the wastewater was treated by wastewater treatment plants. 77.6% of the treated wastewater from wastewater treatment plants (WWTPs) was directly discharged into rivers and the other 22.4% was used to yield reclaimed water. The rate of reclaimed water use was 44.9% (4.5 × 10^9^ m^3^) [1]. These figures are increasing as a consequence of government policies [2,3]. The safety of WWTP effluent has attracted scrutiny, even after it has met the criteria for discharge or reuse [4,5,6]. However, there are still persistent residual chemicals, such as endocrine-disrupting compounds (EDCs), pesticides, and pharmaceutical and personal care products [5,7,8], which are characterized by low concentrations (ng/L), high variety, and complicated physicochemical properties [9].

Endocrine-disrupting activity caused by natural steroid estrogens, including estrone (E1), 17β-estradiol (E2), 17*α*-ethinylestradiol (17α-E2), estriol (E3), and synthetic estrogen (EE2), has been widely detected in the effluent of WWTPs and in their receiving water bodies, as well as in reclaimed water using the effluent as a water source [4,10,11,12]. The levels varied from concentrations of pg/L to μg/L. Due to their potential health risk, adsorption, biodegradation, advanced oxidation process, and photodegradation are frequently used for removing estrogens. Adsorption is considered as the most efficient and economical method.

Abundant oxygen-containing functional groups (such as hydroxyl, carboxyl, and epoxy groups) on the surface of graphene oxide (GO) make it extremely hydrophilic and gives it the capability to be used in aqueous environments as a superior sorbent for removing various pollutants, such as metal ions [13,14], tetracycline antibiotics [15], microcystin [16], and polycyclic aromatic hydrocarbons [17]. However, due to its high dispersibility, it is difficult to separate from the aqueous solution, which may lead to secondary pollution [18,19]. In recent years, magnetic materials have been widely used in the water and wastewater treatment for removing of various pollutants since they can be efficiently separated by magnetic separation technology [20,21,22,23,24,25]. Magnetic graphene oxide (MGO) combines the easy separation of magnetic particles and the high adsorption capacity of GO. In recent years, MGO was reported as a superior sorbent for the removal of antibiotics [26], dyes [27], and metal ions [28,29] with concentrations up to hundreds of mg/L. However, to our knowledge, there is an absence of information focused on the removal of a low level of estrogens with MGO.

The objective of this study was to investigate the adsorption efficiency of MGO for five typical estrogens in both ultrapure and reclaimed water at a ppb level. MGO was synthesized using a chemical coprecipitation method. Estrogens with different concentrations were mixed as samples for investigating MGO adsorption efficiency. Various factors influence the adsorption process, such as pH and temperature, and these were studied. The current study will help to address some of the knowledge gaps about the removal of estrogenic hormones in reclaimed water.

## 2. Materials and Methods

### 2.1. Materials

Analytical standards of E1, E2, 17α-E2, EE2, E3, and acetonitrile were purchased from Aladdin (Shanghai, China). Flake graphite (99.95%, 325 meshes) was provided by Jinrilai Co., Ltd. (Qingdao, China). NH_4_Fe(SO_4_)_2_·12H_2_O and FeCl_2_·4H_2_O were purchased from Sinopharm Chemical Reagent Co., Ltd. (Shanghai, China). CH_2_Cl_2_, C_6_H_14_, Sulfuric acid (H_2_SO_4_, 98%), KMnO_4_, H_2_O_2_ (30%), ammonia solution (25%), and hydrochloric acid were produced by Beijing Chemicals Corporation (Beijing, China).

### 2.2. Preparation and Characterization of MGO

The synthesis of MGO was fulfilled by an in situ chemical coprecipitation of Fe^3+^, Fe^2+^, and GO. GO was synthesized by a pressurized oxidation method described by Bao et al. [30]. Firstly, 100 mL of GO (5 mg/mL) was sonicated for 30 min to form a stable suspension. Then, 8.33 g NH_4_Fe(SO_4_)_2_·12H_2_O and 1.7 g FeCl_2_·4H_2_O were dissolved in 100 mL of ultrapure water under nitrogen protection, followed by a rapid addition of 10 mL of 25% ammonia. Then, the GO suspension was injected dropwise into the solution while being strongly stirred and the solution was keep at 85 °C for 1 h. The product was collected with a magnet and washed with ethanol and ultrapure water three times, then dried at 65 °C for 12 h.

The prepared MGO was characterized by scanning electron microscopy (SEM) (XL30-ESEM, FEI, Hillsboro, OR, USA), transmission electron microscopy (TEM) (TECNAI F20, FEI, Hillsboro, OR, USA), Fourier transform infrared spectroscopy (FTIR) (Nicolet 6700, Thermo Fisher Scientific, Waltham, MA, USA), and X-ray diffraction (XRD) (D8 ADVANCE, Bruker, Karlsruhe, Germany). Additionally, the zeta potential of MGO was also measured (Nano ZS 90, Malvern, UK).

### 2.3. Adsorption Experiments in Estrogen-Spiked Ultrapure Water

The initial concentrations of five estrogens in the 100 mL solutions were 200 μg/L. The experiment was carried out in a 25 °C thermostatic room. Duplicate 1 mL water samples were taken at regular time intervals of 1, 2, 5, 10, 15, and 30 min for HPLC-MS/MS analysis. All the flasks were kept in the dark and agitated on a shaker at 200 rpm, a speed at which adsorption onto the glassware and stripping can be neglected. The estrogen reduction in the blank tests spiked with 200 μg/L ranged from 1.2 ± 1.1% to 3.3 ± 1.8% in 2 h. Therefore, estrogen reduction due to glassware adsorption, soluble organic matter adsorption, and photodegradation were considered minimal. The MGO concentrations were 0.1 g/L. The effect of initial pH was testing by mixing 0.1 g/L MGO with 200 μg/L estrogen mixture solutions at various pH values (pH of 3–11) for 30 min. The pH solution was adjusted with 0.1 mol/L HCl or NaOH solutions.

### 2.4. Adsorption Experiments in WWTPs Effluent

WWTP effluent samples were taken from three local WWTPs and filtered through a 0.45 μm filter. The filtrate was collected for further use. For adsorption experiments, samples were adjusted to a pH of 5. MGO dosage was at 0.1 g/L and the contact time set as 30 min.

Solid phase extraction (SPE)-HPLC-MS/MS was used to analyze estrogen concentration before and after adsorption. SPE was carried out using the Aqua Trace 899 (GL Science, Kyoto, Japan) automated solid-phase extraction instrument. A 1000 mL sample was consecutively extracted by a C_18_ column (6 mL, 500 mg). The C_18_ column was preconditioned consecutively with 2 mL Milli-Q water, 2 mL acetonitrile, and 2 mL methylene dichloride. The filtered sample was loaded onto the SPE column at a flow rate of 10 mL/min. Then, the column was rinsed with 5 mL Milli-Q water and 5 mL hexyl hydride, followed by column elution with 4 mL hexyl hydride at a flow rate of 3 mL/min and desiccation with nitrogen gas for 1 h. Finally, 3 mL acetonitrile was used to redissolve estrogens using a vortex oscillation system for 5 s. The organic eluent was eventually concentrated down to 0.5 mL under a high purity nitrogen stream in a 40 °C water bath and, within a week, Milli-Q water was added to make 1 mL for HPLC-MS/MS analysis.

### 2.5. HPLC-MS/MS Analysis for Estrogens

In this study, a Shimadzu LC-20AD HPLC system (Shimazu, Kyoto, Japan) consisting of an Eclipse Plus C_18_ column (50 × 2.1 mm, 3.5 μm particle size) (Agilent, Santa Clara, CA, USA) was used for estrogen separation. The mobile phase, with a flow rate of 0.3 mL/min, was composed of acetonitrile-water (45:55, *v*/*v*). The sample injection volume was 20 μL.

Analyses were performed using Qtrap 5500 mass spectrometry (Applied Biosystems Sciex, Toronto, ON, Canada) with a Turbo Ion Spray source. Data acquisition was performed in the negative ion mode, and the optimized parameters were as follows: a source temperature of 120 °C, a desolvation temperature of 380 °C, a capillary voltage of 3.2 kV, a desolvation gas flow of 700 L/h, and a cone gas flow of 80 L/h. Argon (99.999%) was used as the collision gas. Quantitative analysis was performed in the multiple reaction monitoring (MRM) mode. The optimal conditions for MS/MS analysis are listed in the Table 1.

The overall method recoveries for the target analytes were between 82.6% and 113.2%, with a relative standard deviation (RSD) less than 13.4%. The limits of quantification (LOQ) of the target analytes were between 2 and 8 ng/L in the pure water and WWTP effluent.

## 3. Results and Discussion

### 3.1. Characterization of Adsorbent

#### 3.1.1. SEM

**Figure 1:**
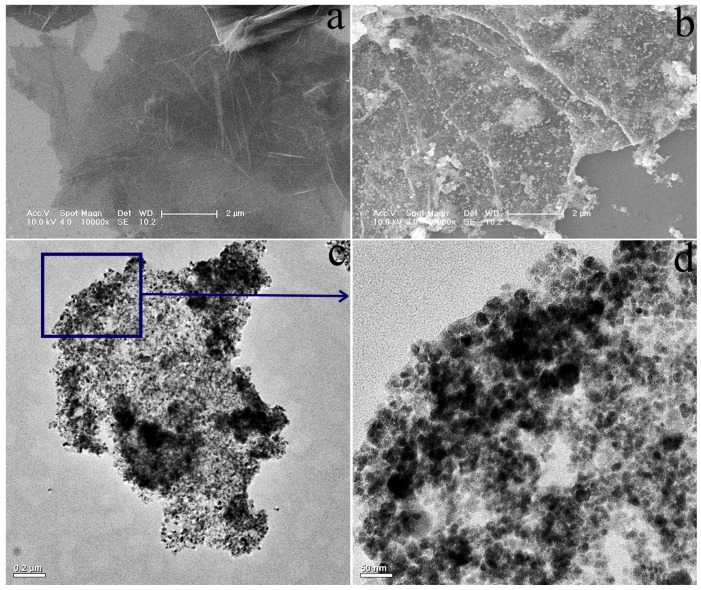
SEM images of (**a**) graphene oxide (GO) and (**b**) magnetic GO (MGO); (**c**,**d**) TEM images of MGO at different resolutions.

**Figure 2:**
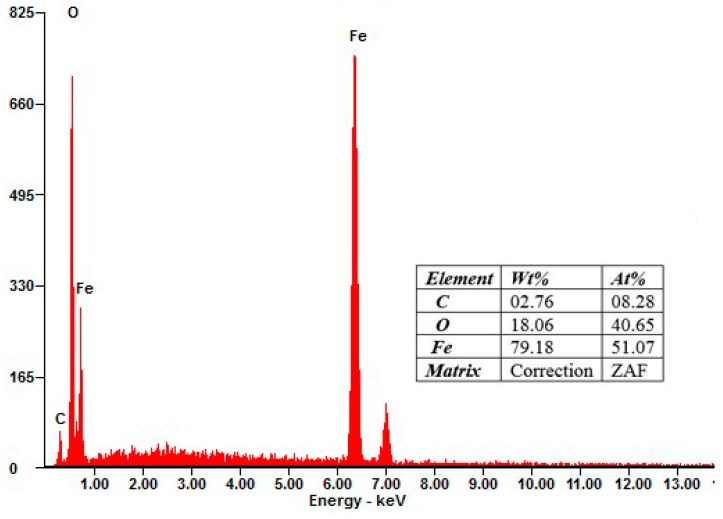
EDX spectrum of MGO.

The morphological structure of MGO was observed using SEM and TEM (Figure 1). Compared with the smooth surface and wrinkles of GO (Figure 1a), it can be seen that Fe_3_O_4_ nanoparticles were successfully coated on the surface of GO to form MGO (Figure 1b). Figure 1c,d show the TEM images of MGO, which suggests that Fe_3_O_4_ nanoparticles with a diameter of about 10–20 nm were well-dispersed on GO sheets. The composition of MGO was verified by energy dispersive X-ray spectroscopy (EDX), as shown in Figure 2. The spectrum showed peaks corresponding to C, O, and Fe. The mass and atom ratio of Fe in MGO was 79.18% and 51.07%, respectively, which also suggested that Fe_3_O_4_ nanoparticles were well-dispersed on GO sheets.

#### 3.1.2. FTIR Spectroscopy

**Figure 3:**
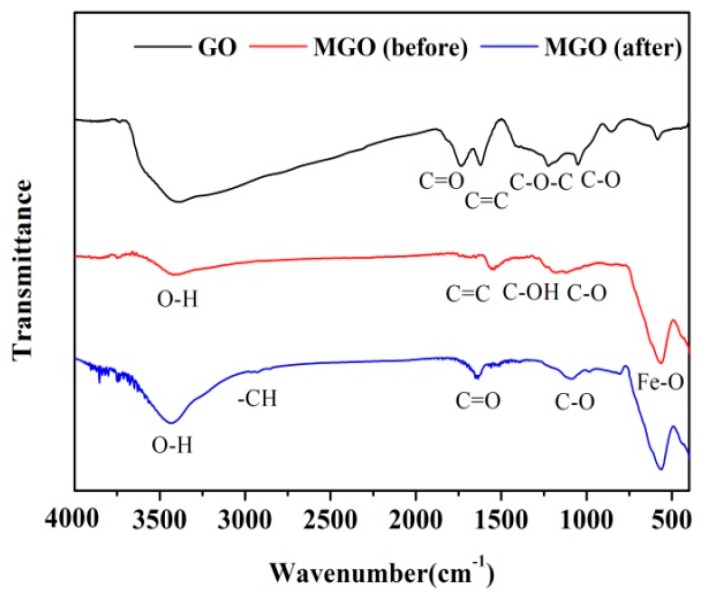
FTIR spectra of GO and MGO before and after the adsorption of estrogens.

FTIR is a valuable technique for understanding the mechanism of adsorption. The FTIR spectra for GO and MGO are shown in Figure 3. For GO, the peak at 1722 cm^−1^ corresponds to the stretching band of C=O in carboxylic acid or carbonyl moieties. The intense peaks at 3431 cm^−1^ are attributed to the stretching of the O–H band. The peak at 1613 cm^−1^ (aromatic C=C) can be assigned to the skeletal vibrations of unoxidized graphitic domains. For the FTIR spectrum of MGO, two new vibrational peaks appear at around 1122 and 1182 cm^−1^. These can be assigned to the formation of either a monodentate complex or a bidentate complex between the carboxyl group and Fe. The appearance of new peaks suggests that Fe_3_O_4_ are covalently bonded to the surface of GO nanosheets. Moreover, the peaks at 561 cm^−1^ can be ascribed to the lattice absorption of Fe_3_O_4_, indicating that Fe_3_O_4_ nanoparticles were loaded onto the surface of GO successfully and the MGO was synthesized successfully.

#### 3.1.3. XRD

**Figure 4:**
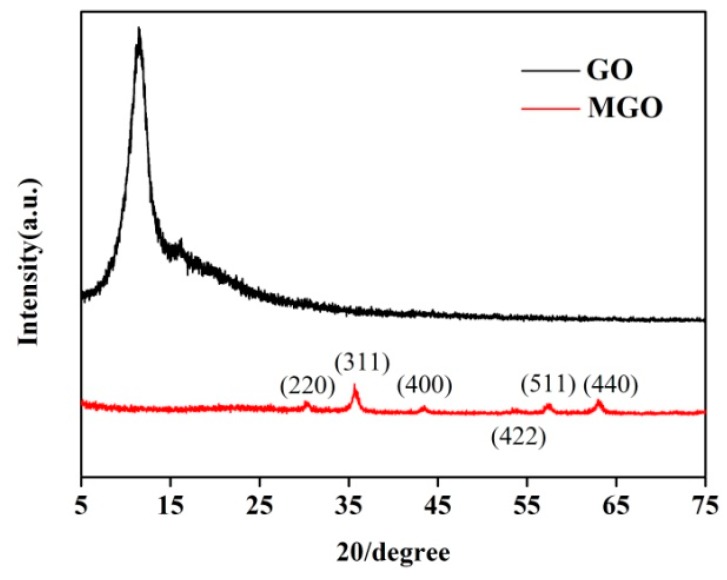
XRD pattern of GO and MGO.

The XRD patterns of the GO and MGO composite are presented in Figure 4. The strongest peaks at 2θ = 11.4° (001) can be appointed to the reflection of the GO, and the peaks at 2θ = 30.3° (220), 35.7° (311), 43.5° (400), 53.9° (422), 57.5° (511), and 63.0° (440) are consistent with the standard XRD data of Fe_3_O_4_. After modification with Fe_3_O_4_, the iron oxides cover up the weak carbon peaks when there is a disappearance of GO at the diffraction peak (2θ = 11.4°). In addition, the presence of magnetite reduces the aggregation of graphene sheets, which results in more monolayer graphene. This, in turn, leads to weaker peaks from carbon being observed [31].

#### 3.1.4. Magnetization

**Figure 5:**
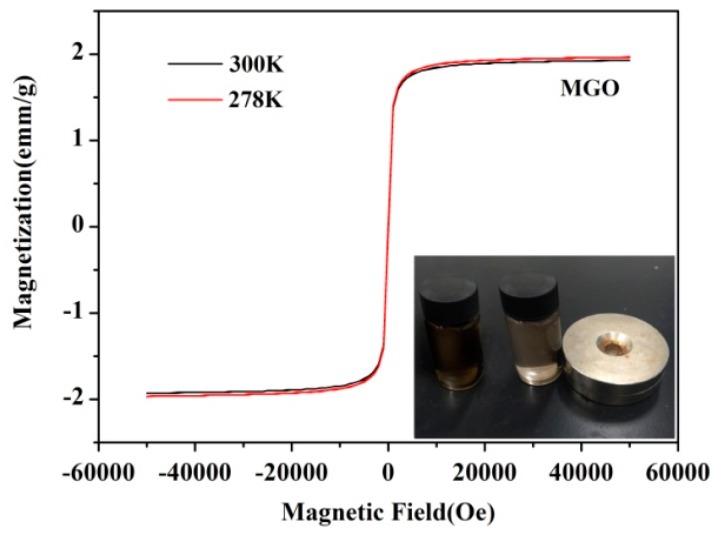
Magnetization curve of MGO. The inset shows magnetic separation of adsorbents from the solution.

Figure 5 represents the “S”-type hysteresis loops of MGO and their magnetization saturation (Ms) at 278 and 300 K, respectively. The Ms of MGO is about 1.93 and 1.97 emu/g at 278 and 300 K, respectively. This evidence demonstrates that the MGO made by coprecipitation synthesis was given stronger magnetization. Though the Ms is not very high, it is enough for magnetic separation, as can be seen from Figure 4. The figure also showed that the effect of temperature on the magnetic of MGO is not significant since the hysteresis loops of MGO at 278 and 300 K tend to coincide. This phenomenon suggests that the variation of temperature does not influence the magnetization of MGO in the subsequent adsorption experiment.

### 3.2. Effect of Initial Solution pH

pH is an essential environmental factor and the most important external factor in influencing the surface charge on the adsorbents and the potential ionization of chemicals. The effect is determined by conducting experiments at initial pH values ranging from 3 to 11. MGO and estrogen are set at 0.1 and 200 μg/L, respectively, and the solutions are unbuffered.

**Figure 6:**
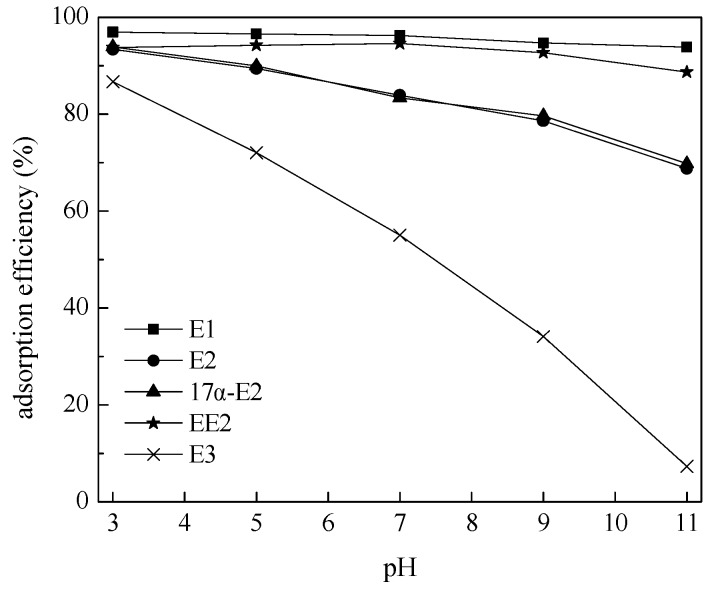
Effect of pH on MGO’s adsorption of estrogens (MGO = 0.05 g, T = 308 K, *t* = 30 min, and estrogens’ concentrations = 200 μg/L).

Figure 6 shows the effect of pH on the MGO adsorption of estrogens. Overall, the sorption decreased with the increasing pH values. E3 is the most sensitive to pH conditions compared to the other estrogens. The highest sorption capacity (86.7%) of E3 was observed at pH 3, while only 7.3% was observed at pH 11. However, aqueous phase pH showed a negligible influence on E1 and EE2 sorption onto MGO. More than 90% of the sorption capacity was obtained at pH 11.

**Figure 7:**
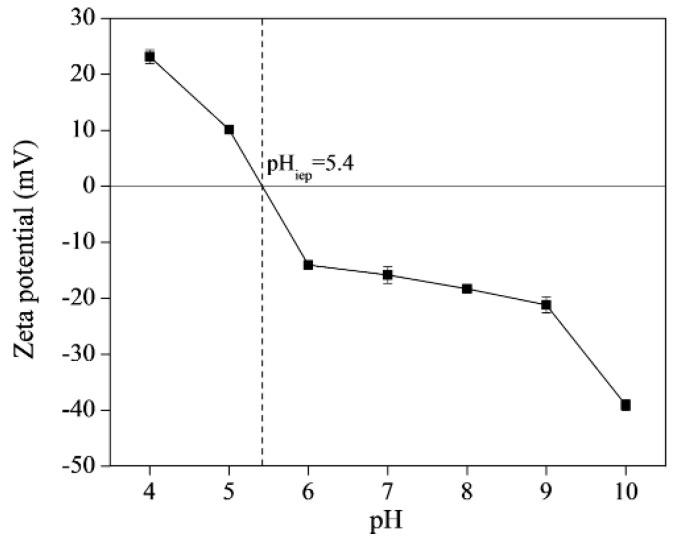
Zeta potential of MGO at different pH values.

Lower estrogen sorption efficiency was observed at higher pH levels, especially basic pH conditions. This might be attributed to the increase in hydroxyl ions, leading to the formation of aqua complexes which retard the sorption phenomena [32]. On the other hand, the benzene ring and phenol hydroxyl of estrogen’s molecular structure were more reactive at acidic conditions and more easily accepting of electrons. This results in the benzene ring rupture further oxidizing to form carboxylic acid functional groups [33], which have a greater affinity to MGO. As shown in Figure 7, the zeta potentials of MGO were negative when pH > 5.4, and the negative charge was enhanced with increasing pH. The repulsive electrostatic interaction established between the negative surface charge of MGO and the estrogens might lead to the lower adsorptive capacity the higher pH ranges. On the other hand, when pH < 5.4, the electrostatic attraction will play a major role in the adsorption of estrogens to positively charged surfaces of MGO.

The sorption efficiency of estrogens onto MGO followed the order of E1 ≈ EE2 > E2 ≈ 17α-E2 > E3 at all the pH ranges, especially at alkaline conditions. This may be attributed to the quantity of the hydroxyl group contacted in estrogen’s molecular structure. There are three hydroxyl groups in the E3 molecule, two in E2 and 17α-E2, and only one in E1 and EE2. More hydroxyl groups lead to a higher negative surface charge of the estrogen molecule, especially in alkaline conditions. Thus, stronger repulsive electrostatic interactions occurred.

### 3.3. Adsorption Kinetics

Pseudo-first-order and pseudo-second-order models (expressed as Equations (1) and (2), respectively) were employed to describe the kinetics of adsorption:

(1) $$\ln\left(q_{e}-q_{t}\right)=\ln q_{e}-k_{1}t$$

(2) tqt=1k2qe2+tq2

where *q_e_* and *q_t_* are adsorption capacity (mg/g) at equilibrium and at time *t* (min), respectively, and *k*_1_ and *k*_2_ are the pseudo-first-order constant (min^−1^) and the pseudo-second-order rate constant (g/(μg∙min)), respectively.

**Figure 8:**
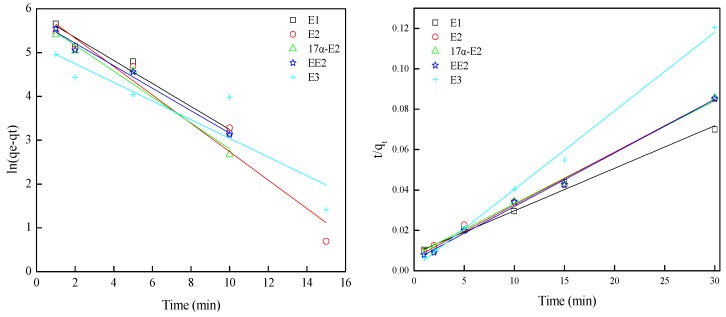
Plots of pseudo-first-order and pseudo-second-order kinetic models.

The kinetic parameters for the two models were determined and listed in Table 2. The results show that the correlation coefficient (*r*) for the pseudo-first-order model is relatively low, and there is a large difference between the calculated adsorption capacity (*q_e_* (cal)) and the experimental value (*q*_exp_), especially for E1, EE2, and E3. The plots of pseudo-first-order and pseudo-second-order kinetic models are shown in Figure 8. As shown in the figure, experimental data had a much better fit with the pseudo-second-order kinetic model. The coefficient factor for this model is very high (*r* > 0.97), and the calculated adsorption capacity agrees well with experimental values.

### 3.4. Effect of Temperature

Temperature is another essential and important external factor which can significantly influence adsorption ability. The effect is determined by conducting experiments at 5, 15, 25, and 35 °C. The solution’s initial pH set at 5. MGO and estrogen are 0.1 g/L and 200 μg/L, respectively.

**Figure 9:**
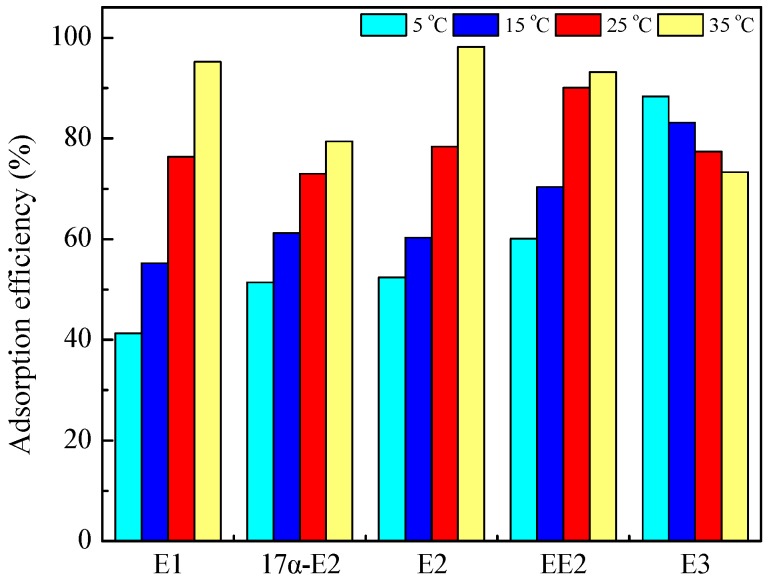
Effect of temperature on the MGO adsorption of estrogens (MGO = 0.05 g, pH = 5, *t* = 30 min, and estrogens’ concentrations = 200 μg/L).

Figure 9 shows the effect of temperature on the MGO adsorption of estrogens. Overall, temperature significantly influences adsorption efficiency. The adsorption efficiency varied from 41.3% to 95.2% for E1, 51.4% to 79.4% for E2, 52.4% to 98.2% for 17α-E2, 60.1% to 93.2% for EE2, and 88.4% to 73.3% for E3. Except for E3, the sorption efficiency was increased with the increasing temperature, which was consistent with previous works [34,35,36]. However, E3 expresses a different trend related to temperature. Lower temperatures seem favorable for the sorption of E3, which is inconsistent with previous work where activated carbon was used as an adsorbent for removing E3 from aqueous solutions [32].

### 3.5. Regeneration and Reusability

**Figure 10:**
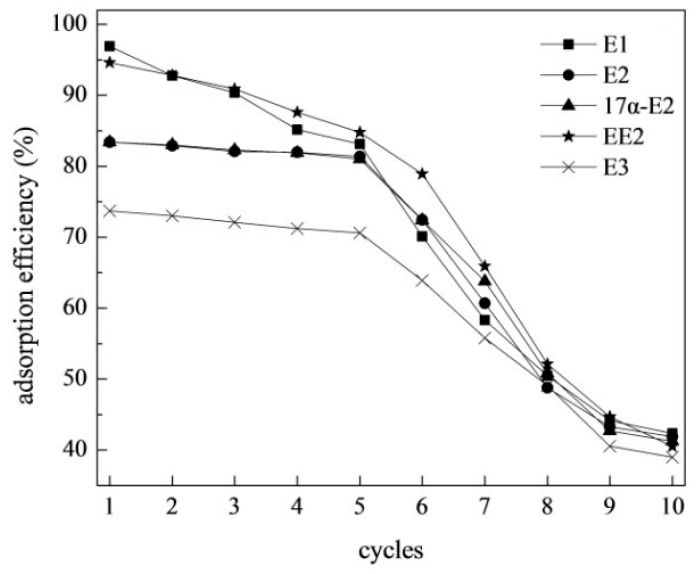
The efficiency of regenerated and reused MGO to adsorption of estrogens.

To study the reusability of MGO, the particles were separated after the adsorption process by using a magnet. Estrogens were desorbed by stirring in 10 mL ethanol for 30 min, and the MGO were dried naturally at room temperature. The recycled adsorbents were used for the next adsorption runs. The results of recycling the experiment for 10 cycles are shown in Figure 10. It is observed that the removal efficiency of all the five estrogens decreased slowly with the increasing regeneration cycles in the first five cycles and dramatically decreased after five cycles. With E1, for example, it is observed that about 96% of the E1 was removed after the first cycle. More than 85% removal efficiency was observed after the 5th cycle, but only 42% was observed after the 10th cycle. These results demonstrated that MGO could be regenerated effectively by ethanol and has the potential for reusability in about five cycles.

### 3.6. Application to WWTP Effluent

WWTPs are considered a significant source of estrogen into the receiving environment [11]. Thus, three WWTP effluent samples were collected from three local WWTPs to investigate the removal of estrogens by MGO. The samples were adjusted to a pH of 5. MGO dosage was 0.1 g/L and the contact time set as 30 min. The concentration of estrogens before and after adsorption are shown in Table 2.

As shown in Table 3, though wastewater was treated by traditional bioprocess, dozens of ng/L estrogens were detected in local WWTPs effluent. Overall, MGO exhibits an effective ability for adsorption removal of estrogens from WWTP effluent. After adsorption, E2 was completely removed from all three samples. More than 90% of E1, 17α-E2, and EE2 were also removed. Just like in pure water, E3 was found to be relatively recalcitrant to MGO in WWTP effluent. More than 4.8 ng/L of E3 was detected in all the three samples after adsorption, which means more than 30% of the E3 remained in samples. The above experiments demonstrated that though the WWTP effluent matrix is much more complex than that of pure water and the estrogen concentration is of low magnitude, MGO can effectively sorb the trace estrogens. This indicates that the MGO has potential for practical applications to remove lower levels of estrogens from real water matrixes.

## 4. Conclusions

In summary, MGO was successfully synthesized using the in situ chemical coprecipitation method. MGO can efficiently remove five typical estrogens from both ultrapure water and WWTP effluent at ppb levels. Sorption was decreased with increasing pH values, and E3 is the most sensitive to pH conditions compared to the other estrogens. Acidic and neutral conditions are favorable for estrogen adsorption onto MGO. Experimental data fit better with the pseudo-second-order kinetic model. Except for E3, sorption efficiency was increased with increasing temperatures. E3 expresses the opposite trend. MGO can be easily separated by using a powerful magnet and regenerated using 10 mL of ethanol. MGO exhibits an effective ability to adsorb the removal of estrogens from WWTP effluent. More than 90% of E2, E1, 17α-E2, and EE2 can be removed.

## Figures and Tables

**Table 1:** Main mass fragments of the target compounds.

Compound	Precursor Ion	Product Ion	Declustering Potentials (V)	Collision Energy (eV)
E1	269.5	145.1	−70	−52
E2	270.8	145.1	−70	−58
17α-E2	270.8	145.1	−70	−58
EE2	294.9	145.1	−60	−53
E3	286.6	145.1	−70	−58

**Table 2:** Kinetic parameters for pseudo-first-order and pseudo-second-order models.

Kinetic Model	Estrogen	*k*_1_ (min^−1^)	*k*_2_ g/(μg∙min)	*q_e_* (cal) (µg/g)	*q_exp_* (µg/g)	*r*
pseudo-first-order	E1	0.436		633.9	387.6	0.94
	E2	0.324		394.6	333.6	0.97
	17α-E2	0.295		317.4	333.6	0.98
	EE2	0.470		625.7	378.4	0.93
	E3	0.213		176.4	294.8	0.88
pseudo-second-order	E1		0.553	400.0	387.6	0.97
	E2		1.001	386.1	333.6	0.99
	17α-E2		1.112	375.9	333.6	0.99
	EE2		0.902	442.5	378.4	0.99
	E3		3.877	289.8	294.8	0.97

**Table 3:** Removal of estrogens in wastewater treatment plant (WWTP) effluent by using MGO.

Estrogens	Samples	Concentration (ng/L)	
		Before Adsorption	After Adsorption
E1	A	31	1.5
	B	56	n.d.
	C	38	n.d.
17α-E2	A	27	n.d.
	B	16	0.8
	C	21	n.d.
E2	A	39	n.d.
	B	17	n.d.
	C	46	n.d.
EE2	A	25	2.3
	B	13	n.d.
	C	27	1.1
E3	A	16	5.8
	B	20	7.9
	C	14	4.8
